# Pattern of extra‐diaphragmatic respiratory muscle activity during exercise in patients with unilateral diaphragm dysfunction

**DOI:** 10.14814/phy2.70635

**Published:** 2025-10-30

**Authors:** Antenor Rodrigues, Zafeiris Louvaris, Sauwaluk Dacha, Mayra Caleffi Pereira, Tin Gojevic, Michele R. Schaeffer, Luc Janssens, Wim Janssens, W. Darlene Reid, Ghislaine Gayan‐Ramirez, Dries Testelmans, Laurent Brochard, Rik Gosselink, Daniel Langer

**Affiliations:** ^1^ Keenan Centre for Biomedical Research Li Ka Shing Knowledge Institute, Unity Health Toronto Toronto Ontario Canada; ^2^ Interdepartmental Division of Critical Care Medicine University of Toronto Toronto Ontario Canada; ^3^ École de Réadaptation, Faculté de Médecine Université Laval Quebec Quebec Canada; ^4^ Centre de Recherche Institut Universitaire De Cardiologie Et De Pneumologie de Québec (IUCPQ) Quebec Quebec Canada; ^5^ Department of Rehabilitation Sciences, Faculty of Movement and Rehabilitation Sciences, Research Group for Rehabilitation in Internal Disorders KU Leuven Leuven Belgium; ^6^ Respiratory Rehabilitation and Respiratory Division University Hospital Leuven Leuven Belgium; ^7^ Pulmonary Division, Heart Institute (InCor), Hospital das Clínicas HCFM USP, Faculdade de Medicina Universidade de SãoPaulo São Paulo Brazil; ^8^ Department of Electrical Engineering, Faculty of Engineering Technology KU Leuven Leuven Belgium; ^9^ Department of Physical Therapy University of Toronto Toronto Ontario Canada; ^10^ KITE, Toronto Rehabilitation Institute University Health Network Toronto Ontario Canada; ^11^ Laboratory of Respiratory Diseases and Thoracic Surgery (BREATHE), Department of Chronic Diseases and Metabolism (CHROMETA) KU Leuven Leuven Belgium

**Keywords:** diaphragm, exercise, physiopathology, respiratory muscles

## Abstract

Whether extra‐diaphragmatic respiratory muscle output is altered in patients with unilateral diaphragm dysfunction (UDD) remains unclear. We compared respiratory pressures and muscle activity during symptom‐limited cardiopulmonary exercise testing (CPET) in 10 patients with UDD and 10 matched controls. Ventilatory variables, dyspnea, and electromyography (EMG) of the diaphragm, scalene, sternocleidomastoid, and parasternal intercostals were assessed at iso‐ventilation and peak ventilation. Compared to controls, patients with UDD showed lower peak workload and oxygen consumption (*p* < 0.029), with a 10% lower but not statistically different peak ventilation (*p* = 0.078). At peak, they were more likely to report high dyspnea (*p* = 0.050). EMG patterns were similar between groups at iso‐ventilation (40 L/min). At peak, controls had greater diaphragm (*p* = 0.007) and parasternal intercostal (*p* = 0.042) activity, while UDD patients showed earlier activation of scalene (*p* = 0.021) and sternocleidomastoid (*p* = 0.049), and greater expiratory muscle recruitment (*p* = 0.004). These findings suggest that patients with UDD rely on earlier and increased activation of extra‐diaphragmatic and expiratory muscles to compensate for reduced diaphragm function during intense exercise. Targeting these compensatory mechanisms may be beneficial in managing diaphragm dysfunction.

## 1 INTRODUCTION

Unilateral diaphragm dysfunction (UDD), the impairment or paralysis of one hemidiaphragm, can lead to compromised pulmonary function, reduced ventilation, and heightened breathlessness in various postures and during physical exertion (Bonnevie et al., 2018; Caleffi Pereira et al., 2021; Elefteriades et al., 2008; Hart et al., 2002; Lisboa et al., 1986; McCool et al., 2018; Puchongmart et al., 2023). While the clinical manifestations of UDD are well documented, much less is known about how other respiratory muscles adapt when ventilatory demand increases. In particular, the physiological behavior of extra‐diaphragmatic respiratory muscles (both inspiratory and expiratory) during exercise in UDD has not been thoroughly investigated in humans. Previous human studies have largely inferred compensatory muscle activation from breathing pattern changes or have focused on the amplitude of extra‐diaphragmatic inspiratory muscle activation, without direct measurements of muscle activation timing during exercise (Caleffi Pereira et al., 2021). As a result, the underlying adaptations in these muscles during physical exertion remain poorly understood in patients with UDD.

Animal models of diaphragm paralysis have provided important insights, demonstrating that when the diaphragm is dysfunctional, extra‐diaphragmatic respiratory muscles increase their activity and adjust their timing to maintain ventilation (De Troyer & Boriek, 2011; Katagiri et al., 1994). For example, in canine models, paralysis of the diaphragm leads to greater activation of parasternal intercostals and abdominal muscles, as well as an early onset of extra‐diaphragmatic inspiratory muscle activity early in the inspiratory cycle to compensate for the loss of diaphragm function (De Troyer & Boriek, 2011; Katagiri et al., 1994). In humans, available evidence is limited. One case study of an adult who developed unilateral diaphragm paralysis found reductions in lung volumes and preserved overall ventilatory responsiveness to CO_2_, but with a notable increase in inspiratory drive (elevated P0.1) after the onset of paralysis, suggesting recruitment of compensatory mechanisms. A recent study in 35 patients with UDD reported that the EMG amplitude of extra‐diaphragmatic inspiratory muscles (scalene and sternocleidomastoid) during exercise was greater in UDD than in healthy controls (Caleffi Pereira et al., 2021). However, that study assessed only inspiratory muscle activation and pressures, without examining expiratory muscle involvement or the timing of muscle recruitment. Thus, while it confirmed an increased neural drive to extra‐diaphragmatic inspiratory muscles in UDD (paralleling observations in animal models), it remains unclear whether the timing of respiratory muscle activation is altered in humans with UDD. Indirect findings hint at such changes: for instance, patients with UDD achieve their peak negative inspiratory pressure (esophageal pressure) more rapidly during inspiration than healthy individuals, which could reflect an altered coordination of muscle recruitment (Bonnevie et al., 2018; Hart et al., 2002; Lisboa et al., 1986). However, this faster pressure generation might result from passive abdominal mechanics (relaxation of the abdominal wall at end‐expiration aiding the diaphragm's next contraction), earlier activation of extra‐diaphragmatic inspiratory muscles at the start of inspiration, or a combination of both mechanisms—as seen in animal models.

Given this gap in knowledge, our objective was to characterize the pattern of respiratory muscle activation in patients with UDD compared to healthy controls during incremental exercise. Specifically, we aimed to (1) determine the onset timing of diaphragm, parasternal intercostal, scalene, and sternocleidomastoid activation relative to inspiratory flow, and (2) assess expiratory muscle pressure generation during exercise. We also compared respiratory pressures, lung volumes, and EMG amplitudes of the diaphragm and extra‐diaphragmatic muscles between groups.

We hypothesized that, compared with healthy controls, patients with UDD would (1) exhibit earlier activation of extra‐diaphragmatic inspiratory muscles relative to inspiratory flow, (2) generate greater expiratory muscle pressure during expiration, and (3) produce lower transdiaphragmatic pressures but maintain near‐normal esophageal pressures during exercise.

## 2 METHODS

### 2.1 Participants

Ten adult patients with unilateral diaphragm dysfunction and 10 age‐, sex‐ and body mass index (BMI)‐matched, apparently healthy, non‐smoking controls were included. The research was approved by the Ethics Committee Research of University Hospital Leuven (S60754). Patients with unilateral diaphragm dysfunction were recruited from the University Hospitals Leuven within 12 months of receiving their diagnosis. The inclusion criteria for patients with unilateral diaphragm dysfunction were a Baseline Dyspnea Index (BDI) ≤9, an elevated hemidiaphragm on the chest radiography, paradoxical diaphragmatic movement during a sniff maneuver on chest fluoroscopy, and either a seated vital capacity <75% of predicted or a reduction in forced vital capacity (FVC) >15% between seated and supine positions or a maximal inspiratory pressure <70% of predicted. The exclusion criteria were malignancy, psychiatric disorders, progressive neurological, neuromuscular or vestibular disorders, cardiorespiratory diseases that could contribute to dyspnea or orthopedic problems that could affect exercise performance.

Apparently healthy, nonsmoking controls were recruited from the Leuven region. The inclusion criteria for the healthy controls were body‐mass index (BMI) <30 kg/m^2^ with normal spirometry (forced expiratory volume in 1 s [FEV_1_] to FVC >0.70 and FEV_1_ ≥ 80% predicted). The exclusion criteria for the healthy controls were the same as those described for the patients with unilateral diaphragm dysfunction.

### 2.2 Study design

This was an ancillary study from a randomized clinical trial that aimed to characterize the effects of a six‐month inspiratory muscle training program on exertional dyspnea and inspiratory muscle function in patients with unilateral diaphragm dysfunction (Schaeffer et al., 2023). All patients and healthy controls provided written informed consent before enrollment in accordance with the Declaration of Helsinki.

After enrollment, patients with unilateral diaphragm dysfunction and healthy controls performed spirometry, measurements of maximum inspiratory strength (MIP) and maximum esophageal, gastric and transdiaphragmatic inspiratory pressures (Pes, Pga, and Pdi, respectively) during a sniff maneuver. Patients with unilateral diaphragm dysfunction rated the severity of their dyspnea according to the Baseline Dyspnea Index (BDI). Additionally, phrenic nerve conduction studies were performed only in patients to characterize and confirm unilateral diaphragm dysfunction.

Next, patients with unilateral diaphragm dysfunction and healthy controls performed an incremental symptom‐limited cardiopulmonary exercise test (CPET) on an electrically braked cycle ergometer (Ergoline 800 s; Duomed, BE) using a 20 W/min stepwise protocol with serial measurements of inspiratory capacity. During the CPET, ventilatory responses (e.g., respiratory flow, tidal volume, respiratory rate, and inspiratory capacity [IC]) were measured by having patients and healthy controls breathe through a mouthpiece connected to a cardiopulmonary testing system (Vmax Vs229; Dumed). ΔIC during the CPET was calculated as: ΔIC = peak IC – rest IC. Pes, Pga, Pdi and electromyography of the crural diaphragm (EAdi) were recorded continuously using a combined multipair esophageal electrode catheter (Guangzhou Yinghui Medical Equipment Ltd., CN). Electrocardiogram (ECG) and surface electromyography (sEMG; Noraxon, Scottsdale, USA) of the parasternal intercostal, scalene and sternocleidomastoid muscles were recorded continuously during the CPET.

The modified Borg scale was used to measure dyspnea intensity and sensation of leg fatigue during the CPET. We calculated the probability that the Borg scale score for dyspnea was abnormally high (Pabnorm) using predictive equations for peak ventilation (L/min) derived from age, sex, and BMI (Ekstrom et al., 2024; Ekstrom, Lewthwaite, & Jensen, 2024). Pabnorm ranges from 0 to 1, with higher values indicating a greater probability that the reported Borg score exceeded the expected normal range. This approach provides a probability‐based assessment rather than relying on a fixed cutoff, thereby offering a more nuanced interpretation of abnormal dyspnea responses.

### 2.3 Data collection

#### 2.3.1 Lung function

Patients with unilateral diaphragm dysfunction and healthy controls performed spirometry (Vmax229d with Vs62j body plethysmograph; Duomed) in a sitting position according to international guidelines (Graham et al., 2019). FVC, FEV_1_, and FEV_1_/FVC and a 10 s maximal voluntary ventilation (MVV) maneuver were quantified and reported in absolute and percent predicted values (Hall et al., 2021). Additionally, only patients with unilateral diaphragm dysfunction performed spirometry in the supine position and the difference between FVC in the supine and sitting positions was calculated.

#### 2.3.2 Phrenic nerve conduction studies

Phrenic nerve conduction studies were performed only in patients with unilateral diaphragm dysfunction to characterize and confirm diaphragm dysfunction. A detailed description of the technique has been published elsewhere (Gayan‐Ramirez et al., 2008). Briefly, each phrenic nerve was stimulated sequentially at the posterior border of the sternocleidomastoid in the supraclavicular fossa above the clavicle applying a square‐wave electrical pulse with a duration of 100 ms. Compound motor action potentials (CMAP) were recorded by two surface electrodes located above the tip of the xiphoid process (active electrode) and at the costal margin at the level of the 7th intercostal space. CMAP latency and amplitude were recorded in both the dysfunctional and preserved hemidiaphragm. CMAP latency was calculated as the time from the stimulus artifact to the beginning of the deflection in the CMAP signal in milliseconds. The CMAP amplitude was calculated as the maximum value in μV achieved during the CMAP.

CMAP latency and amplitude from the dysfunctional hemidiaphragm were used to confirm unilateral diaphragm dysfunction diagnosis. A CMAP latency greater than 8.1 ms and an amplitude less than 300 μV were used as threshold values.

#### 2.3.3 Respiratory muscle strength, pressures and electrical activity of the respiratory muscles

Pes, Pga, and EAdi were measured by a combined multipair esophageal electrode catheter with esophageal and gastric balloons (Guangzhou Yinghui Medical Equipment Ltd., CN) inserted nasally after topical anesthesia and positioned in accordance with established methodology as previously reported (Langer et al., 2018; Rodrigues et al., 2020).

The electrical activity of the parasternal intercostal (EApi), scalene (EAsca), and sternocleidomastoid (EAscm) was collected using sEMG (Noraxon, Scottsdale, USA). sEMG electrodes were placed on the right hemithorax: (i) over the parasternal intercostal muscle (EApi) at the parasternal space between the 2nd and 3rd rib lateral to the sternum; (ii) over the scalene (EAsca) on the posterior triangle of the neck at the level of the cricoid process; (iii) along the long axis of the sternocleidomastoid (EAscm) at its midpoint between the mastoid process and the medial aspect of the clavicle.

Esophageal and gastric balloons were filled with 0.8 and 1.4 mL of air, respectively. Pes and Pga were sampled at 100 Hz using differential pressure transducers (Micro1401‐3, Cambridge Electronic Design Limited, Cambridge, UK). EAdi and sEMGs were sampled at 2000 Hz (Micro1401‐3, Cambridge Electronic Design Limited, Cambridge, UK) and amplified (Biomedical amplifier, Guangzhou, China). Ventilatory responses, Pes, Pga, EAdi, and sEMGs were time‐aligned and recorded using data acquisition software (Spike 2, Cambridge Electronic Design Limited, Cambridge, UK) and stored for offline analysis (see below).

MIP was measured at residual volume using a mouth piece according to international guidelines (MicroRPM; Micromedical, UK) (Laveneziana et al., 2019). Maximum inspiratory voluntary Pes, Pga, and Pdi were measured during sniff maneuvers.

### 2.4 Offline data analysis

#### 2.4.1 Respiratory muscle pressures

Pdi was calculated as the difference between Pga and Pes. We calculated inspiratory Pes, Pga, and Pdi swings breath‐by‐breath as the difference between the average pressures generated during inspiration and expiration. The Pes swing (∆Pes) was used as an index of global inspiratory muscle effort whereas the Pdi swing (∆Pdi) was used as an index of diaphragmatic effort (Laveneziana et al., 2019). ∆Pes and ∆Pdi were normalized by the maximum value achieved during the sniff maneuvers (∆Pes, sniff and ∆Pdi, sniff, respectively) as an index of relative effort. We further calculated the Pga rise, a surrogate for expiratory muscle use, as the difference between the highest Pga during expiration and the minimum Pga during inspiration. While Pga swings can result from both diaphragmatic contraction during inspiration and expiratory muscle contraction during expiration, the Pga rise specifically reflects expiratory muscle contraction during expiration. Inspiration and expiration were identified based on the crossing point of the flow signal from positive to negative that was collected during the CPET in synchrony with the Pes and Pga.

#### 2.4.2 EAdi and sEMG offline analysis

We used a semiautomated algorithm previously developed and validated by our group to analyze the EAdi and sEMGs (Dacha et al., 2019; Rodrigues et al., 2021; Spasojevic et al., 2021). A detailed description is provided elsewhere. Briefly, the raw EAdi and sEMG were imported into the LABVIEW software (National Instrument, Austin, TX, USA). Several steps were carried out before the onset timing of the diaphragm, parasternal intercostal, scalene and sternocleidomastoid electrical activity is detected breath‐by‐breath. First, a bidirectional high‐pass filter at 5 Hz 2nd order Butterworth was applied for removing motion artifacts from the EAdi and sEMGs signals. Next, we used a least mean square adaptive filter for removing ECG artifacts which is a pattern recognition method that uses a time‐aligned ECG signal to remove the ECG frequency content from the EAdi and sEMGs signals while minimizing alteration of the EMG signals. Next, the root mean square (RMS) of the EAdi and each muscle sEMGs were calculated breath‐by‐breath. Each breath was identified based on the zero‐crossing point of the flow signal from positive to negative collected during the CPET in synchrony with the EAdi and sEMGs and imported into the LABVIEW software. The mean RMS during inspiration of the EAdi and of each muscle sEMGs was calculated from their ECG‐filtered signals as a surrogate of the amplitude of the muscle electrical activity. EAdi and sEMG RMS were normalized by the maximum activation achieved during inspiratory capacity maneuvers performed during the CPET.

Then, the onset timing of the EAdi and each muscle's sEMGs was defined breath‐by‐breath by the rise of the RMS of the EAdi or sEMG as previously described (Dacha et al., 2019; Rodrigues et al., 2021). Using the first derivative function of the EAdi and of each muscle's sEMGs, the rising and descending phases of their RMS are identified. The onset time of each muscle's electrical activity was defined as the timepoint of the 5% rise of the EAdi or sEMGs RMS of their respective maximums (±1 msec). This 5% threshold avoids mistakenly identifying inherent variability of the baseline EMG signal as an onset of the muscle's electrical activity. The EAdi or each muscle's sEMG onset timing was reported as negative or positive based on whether it occurred before or after the onset of the inspiratory flow signal, respectively. The timing difference (dP) between the onset timing of the EAdi or each muscle's sEMG and the onset of the inspiratory flow was reported in absolute values (ms) and normalized by the inspiratory time (%Ti). Time alignment between flow, pressure and EMG signals in Spike was tested with an external trigger and adjusted by an Engineer.

### 2.5 Statistical analysis

Continuous data were expressed as median [25%–75% IQR] and categorical variables as absolute numbers and percentages. The distribution of sex between the groups was assessed with the chi‐squared test. We compared Pes, Pga and Pdi swings, Pga rise, ventilatory responses, respiratory muscle strength and respiratory muscle's electrical activity at iso‐ventilation and peak ventilation between patients with unilateral diaphragm dysfunction and healthy controls using Student's *t*‐test. Iso‐ventilation was defined as the highest equivalent ventilation in L/min that was attained by all patients with unilateral diaphragm dysfunction or healthy control during the CPET. Peak ventilation was defined as the last 60 s of the CPET. *p* Value ≤0.05 was used as the threshold for statistical significance.

## 3 RESULTS

### 3.1 Healthy controls and patients with unilateral diaphragm dysfunction characteristics

Ten patients with unilateral diaphragm dysfunction and 10 healthy controls were included (Table 1). Both groups were similar regarding age, sex, and BMI (*p* ≥ 0.140 for all). The severity of breathlessness according to BDI for patients with unilateral diaphragm dysfunction was moderate (BDI score: 7 [7–9]). Patients with unilateral diaphragm dysfunction had reduced FEV_1_ and FVC (percent of predicted) compared to healthy controls (*p* ≤ 0.001 for all). Patients with unilateral diaphragm dysfunction had reduced MIP in percent predicted (*p* = 0.007). Inspiratory Pes, Pga, and Pdi sniff were reduced in patients with unilateral diaphragm dysfunction compared to healthy controls (*p* ≤ 0.040 for all). Diaphragm dysfunction occurred on the left side in 40% of the patients with unilateral diaphragm dysfunction (Table 1). In our UDD patients CMAP latency was 12 ± 4 ms, which is greater than the threshold of 8.1 s, and amplitude was 87 ± 25 μV, which was below the normal threshold values, in all patients with unilateral diaphragm dysfunction.

**TABLE 1:** Characteristics of healthy controls and patients with unilateral diaphragm paralysis.

	Controls (*n* = 10)	UDD (*n* = 10)
Demographics		
Sex, male *n* (%)	7 (70%)	6 (60%)
Age, years	55 [50 to 60]	59 [56 to 64]
Weight, kg	79 [71 to 84]	83 [75 to 95]
Height, cm	174 [169 to 176]	172 [166 to 182]
BMI, kg/m^2^	26.0 [23.7 to 28.2]	27.5 [26.5 to 29.4]
Side of dysfunction, left *n* (%)	NA	4 (40%)
Pulmonary function		
FVC, % predicted	105 [99 to 112]	68 [65 to 75]*
FEV_1_, % predicted	93 [86 to 106]	63 [54 to 67]*
FEV_1_/FVC, ratio	0.74 [0.71 to 0.78]	0.66 [0.63 to 0.70]
FVC_supine_ – FVC_seated_, %∆	NA	−27 [−32 to −23]
IC, L	3.18 [2.85 to 3.77]	2.62 [2.44 to 3.17]
Respiratory muscle strength		
MIP, cmH_2_O	111 [104 to 121]	101 [87 to 112]
MIP, % predicted	116 [100 to 136]	91 [79 to 100]*
Sniff Pes, cmH_2_O	−69 [−88 to −61]	−53 [−67 to −50]*
Sniff Pga, cmH_2_O	30 [12 to 36]	−4 [−10 to 3]*
Sniff Pdi, cmH_2_O	103 [96 to 108]	48 [44 to 56]*
Cardiorespiratory performance metrics		
VO_2_, mL/kg/min	36.1 [35.6 to 37.5]	27.1 [23.0 to 30.8]*
VO_2_, % predicted	121 [112 to 143]	99 [90 to 115]
Load, Watts	230 [190 to 278]	180 [140 to 200]*
HR, bpm	160 [153 to 170]	130 [121 to 133]*

*Note*: Data is expressed as median [25%–75%IQR] for continuous variables and absolute numbers and percentages for categorical variables.

Abbreviations: BMI, body mass index; BDI, baseline dyspnea index; FEV_1_, forced expiratory volume in the first second; FVC, forced vital capacity; MIP, maximal inspiratory pressure; Pdi, transdiaphragmatic pressure; Pes, esophageal pressure; Pga, gastric pressure.

* *p* < 0.05.

### 3.2 Peak exercise capacity

At peak ventilation, patients with unilateral diaphragm dysfunction achieved lower absolute peak workload (Table 1; *p* = 0.028), maximum oxygen consumption in both absolute (Table 1; *p* = 0.003) and percent predicted (Table 1; *p* = 0.003) and heart rate (Table 1; *p* = 0.013) compared to healthy controls. At peak ventilation, dyspnea intensity measured by the Borg scale was similar between patients with unilateral diaphragm paralysis and healthy controls (8 [7–10] vs. 7 [6–9], respectively; *p* = 0.360). However, after adjusting the Borg scale by ventilation (L/min), patients with unilateral diaphragm paralysis had a *p*_abnorm_ greater than healthy controls (0.89 [0.86–0.94] vs. 0.66 [0.34–0.80]; *p* = 0.050).

### 3.3 Ventilatory responses

Iso‐ventilation was 40 L/min, corresponding to a higher percentage of the maximum voluntary ventilation for patients with unilateral diaphragm dysfunction than healthy controls (46% [40%–60%] vs. 29% [28%–31%], respectively; *p* = 0.034). Peak ventilation corresponded to 71% [65%–87%] and 67% [55%–80%] of the maximum voluntary ventilation for patients with unilateral diaphragm dysfunction and healthy controls, respectively (*p* = 0.210). There were no statistically significant differences in tidal volume, respiratory rate, inspiratory time, duty cycle, ΔIC or inspiratory peak flow between patients with unilateral diaphragm dysfunction and healthy controls either at iso‐ventilation or peak ventilation (Table 1; *p* ≥ 0.130 for all).

### 3.4 Respiratory muscle pressure responses

**FIGURE 1:**
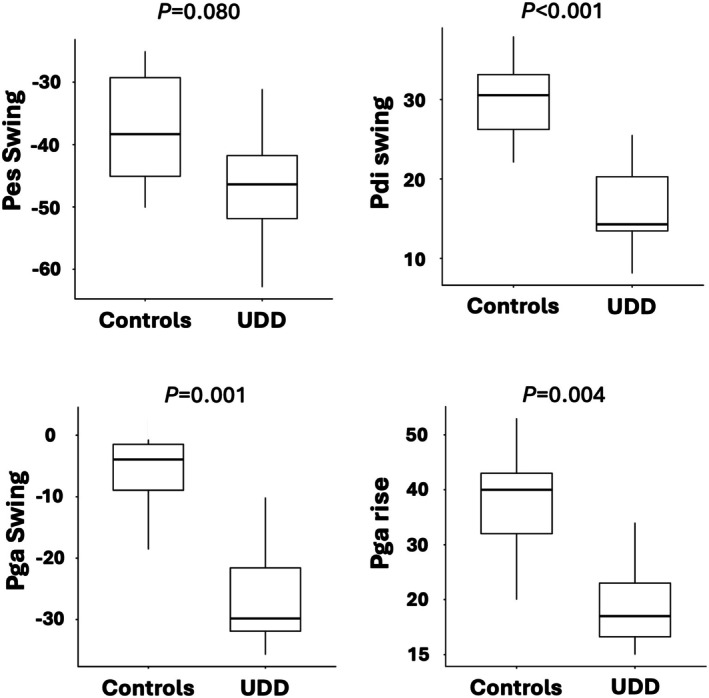
Respiratory pressures at peak ventilation. (a) Pes swing; (b) Pdi swing; (c) Pga swing; (d) Pga rise. Pdi, transdiaphragmatic pressure; Pes, esophageal pressure; Pga, gastric pressure. Pes, Pga, and Pdi swings were calculated as the difference between the average pressures generated during inspiration and expiration. Pga rise was calculated as the difference between the highest Pga during expiration and the minimum Pga during inspiration. Data are expressed as median[25%–75% IQR].

At iso‐ventilation, absolute (cmH_2_O) Pga swings were larger (*p* = 0.025) and absolute (cmH_2_O) Pdi swings were smaller (*p* = 0.020) in patients with UDD than controls (Table 2). At peak ventilation, absolute (cmH_2_O) Pga swing (*p* = 0.001) and absolute (cmH_2_O) Pga rise (*p* = 0.004) were larger and absolute (cmH_2_O) Pdi swings were smaller (*p* < 0.001) in patients with UDD than controls (Table 1 and Figure 1).

**TABLE 2:** Comparisons between ventilatory and metabolic responses and respiratory muscle electromyography activity between controls and patients with unilateral diaphragm dysfunction at iso‐ventilation and peak incremental cycling cardiopulmonary exercise test.

	Iso‐ventilation		Peak	
	Controls	UDD	Controls	UDD
Ventilatory variables				
Ve, L/min	40 [39 to 41]	40 [39 to 40]	83 [74 to 94]	74 [58 to 80]
Ve, %MVV	29 [28 to 31]	46 [40 to 60]*	67 [55 to 80]	71 [65 to 97]
Vt, L	2.05 [1.65 to 2.22]	1.70 [1.34 to 1.82]	2.59 [1.99 to 3.00]	2.04 [1.50 to 2.11]
Vt/IC, %	60 [52 to 65]	60 [57 to 65]	76 [73 to 85]	71 [66 to 78]
RR, bpm	20 [18 to 22]	23 [22 to 31]	32 [30 to 37]	38 [34 to 41]
Ti, sec	1.31 [1.18 to 1.47]	1.15 [0.90 to 1.25]	0.89 [0.74 to 0.92]	0.76 [0.71 to 0.83]
Ti/Ttot, %	45 [43 to 46]	46 [45 to 47]	47 [45 to 48]	47 [46 to 49]
Peak flow, L/min	1.95 [1.88 to 2.08]	2.02 [1.97 to 2.17]	4.01 [3.80 to 4.18]	3.29 [2.98 to 3.77]
∆IC, L	−0.09 [−0.25 to 0.14]	0.19 [−0.21 to 0.37]	0.15 [−0.28 to 0.24]	0.29 [−0.15 to 0.48]
Symptoms of dyspnea and leg fatigue				
Borg dyspnea (0–10)	2 [1 to 3]	3 [3 to 4]	7 [6 to 9]	8 [7 to 10]
Borg leg fatigue (0–10)	2 [1 to 3]	3 [3 to 4]	7 [6 to 8]	7 [5 to 8]
EMG RMS %max				
EAdi, normalized RMS	70 [58 to 79]	69 [54 to 75]	105 [96 to 119]	84 [72 to 86]*
EAps, normalized RMS	36 [29 to 40]	31 [30 to 32]	42 [41 to 50]	34 [33 to 35]*
EAsa, normalized RMS	32 [14 to 39]	41 [38 to 49]	37 [19 to 78]	59 [45 to 65]
EAscm, normalized RMS	29 [24 to 31]	31 [27 to 47]	43 [39 to 46]	40 [34 to 54]
Respiratory muscle pressures				
∆Pes, cmH_2_O	−18 [−21 to −14]	−21 [−34 to −20]	−38 [−45 to −29]	−46 [−52 to −42]
∆Pes, %sniff	24 [19 to 31]	43 [32 to 67]	52 [45 to 59]	94 [63 to 106]*
∆Pdi, cmH_2_O	21 [17 to 22]	14 [9 to 16]*	31 [26 to 33]	14 [14 to 20]*
∆Pdi, %sniff	20 [17 to 22]	33 [17 to 34]	28 [26 to 30]	33 [25 to 39]
∆Pga, cmH_2_O	0 [−1 to 1]	−7 [14 to −6]*	−4 [−9 to −1]	−30 [−32 to −21]*
Pga rise, cmH_2_O	8 [7 to 10]	13 [10 to 15]	17 [13 to 23]	40 [32 to 43]*
Cardiorespiratory performance metrics				
Load, Watts	120 [120 to 120]	103 [85 to 120]	230 [190 to 278]	180 [140 to 200]*
HR, bpm	114 [106 to 134]	88 [86 to 107]	160 [153 to 170]	130 [121 to 133]*
HR, %max	68 [64 to 78]	55 [52 to 64]	92 [85 to 100]	78 [77 to 88]
VO_2_, ml/kg/min	23.6 [19.3 to 26.3]	17.2 [16.7 to 18.3]*	36.1 [35.6 to 37.5]	27.1 [23.0 to 30.8]*

*Note*: Data is expressed as median [25%–75%IQR]. We calculate inspiratory Pes, Pga and Pdi swings breath‐by‐breath as the difference between the average pressures generated during inspiration and expiration. ΔIC = peak – rest.

Abbreviations: EAdi, diaphragm electromyography; EAps, parasternal intercostal electromyography; EAsa, scalene electromyography; EAscm, sternocleidomastoid electromyography; MVV, maximum voluntary ventilation; normalized RMS, root mean square normalized by the highest RMS during an inspiratory capacity maneuver; Pdi, transdiaphragmatic pressure; Pes, esophageal pressure; Pga, gastric pressure; RR, respiratory rate; Te, expiratory time; Ti, inspiratory time; Ttot, total time of the respiratory cycle; UDD, unilateral diaphragm dysfunction; Ve, minute ventilation; Vt, tidal volume.

* *p* < 0.05 versus controls.

After normalizing the Pes and Pdi swings to the maximum values achieved during the sniff maneuver (%sniff), normalized Pes swing (%sniff) was greater in patients with UDD than controls (at isoventilation *p* = 0.054 and at peak exercise *p* = 0.028) while no statistically significant differences were observed for Pdi swing (%sniff) at iso‐ventilation (*p* = 0.360) or at peak ventilation (*p* = 0.540) (Table 1).

Pdi during IC was not different between rest, isoventilation and peak (26 [16–28], 19 [15–29], 24 [19–28]; cmH_2_O *p* = 0.215).

### 3.5 Amplitude of the electrical activity of the respiratory muscles

Normalized EAdi, EAps, EAsa, and EAscm were similar in both groups at iso‐ventilation (*p* ≥ 0.270; Table 1). However, at peak ventilation, normalized EAdi (*p* = 0.007) and EAps (*p* = 0.042) were lower in patients with unilateral diaphragm dysfunction than in controls, while normalized EAsa (*p* = 0.670) and EAscm (*p* = 0.690) were similar between both patients with unilateral diaphragm dysfunction and controls (Table 2).

### 3.6 Onset timing of the electrical activity of the respiratory muscles

**FIGURE 2:**
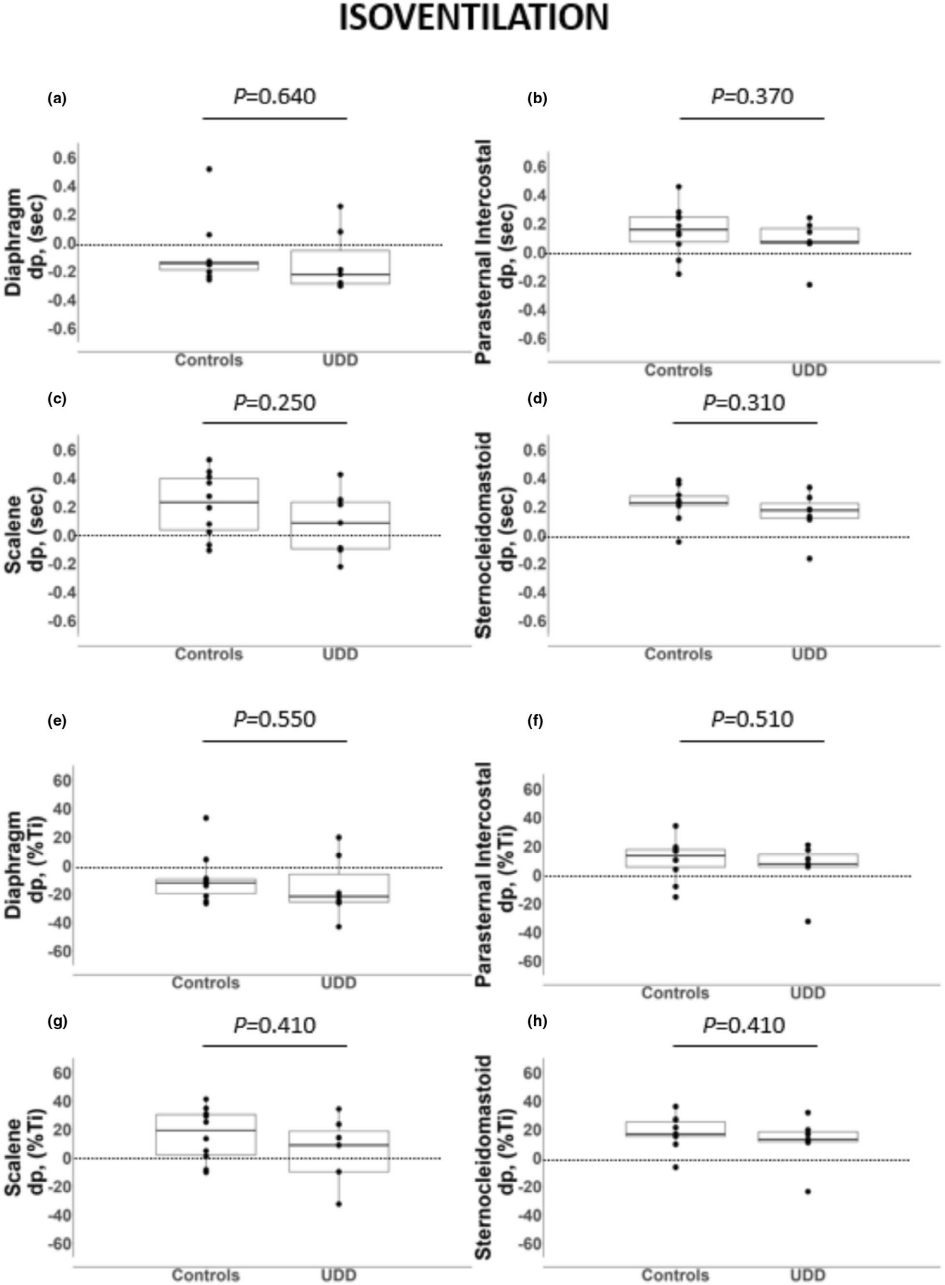
Onset timing of the electrical activity of the diaphragm, parasternal intercostal, scalene and sternocleidomastoid in seconds (a–d) and percent of the inspiratory time (e–h) at iso‐ventilation during the cardiopulmonary exercise test (CPET). Data are expressed as median [25%–75% IQR]. dP, the difference between the onset of the muscle electrical activity and the inspiratory flow; Ti, nspiratory time.

**FIGURE 3:**
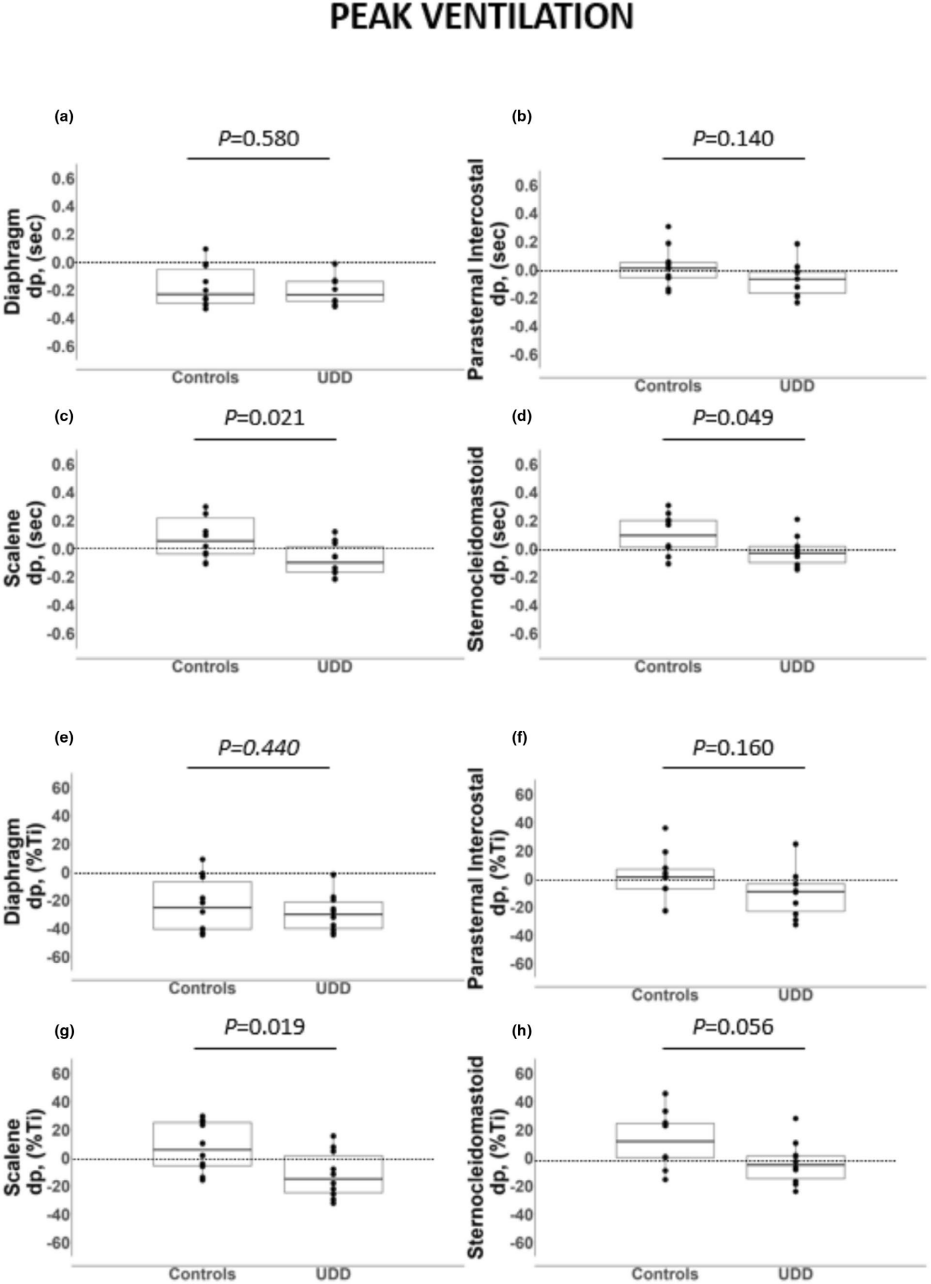
Onset timing of the electrical activity of the diaphragm, parasternal intercostal, scalene, and sternocleidomastoid in seconds (a–d) and percent of the inspiratory time (e–h) at peak ventilation. Data are expressed as median [25%–75% IQR]. dP, the difference between the onset of the muscle electrical activity and the inspiratory flow; Ti, inspiratory time.

At iso‐ventilation, onset timing of the electrical activity of the diaphragm, parasternal intercostal, scalene and sternocleidomastoid was similar between patients with unilateral diaphragm dysfunction and controls (*p* ≥ 0.250 for all; Figure 2). However, at peak ventilation, scalene and sternocleidomastoid onset timing was earlier in patients with unilateral diaphragm dysfunction than in healthy controls (Figure 3; *p* ≤ 0.049).

Only the diaphragm onset timing occurred before the onset of the inspiratory flow in both patients with unilateral diaphragm dysfunction and healthy controls at iso‐ventilation (Figure 2). However, at peak ventilation, diaphragm, parasternal intercostal, scalene, and sternocleidomastoid onset timing occurred before the onset of the inspiratory flow in patients with unilateral diaphragm dysfunction (Figure 3). In contrast, in healthy controls, still only the diaphragm onset timing occurred before the onset of the inspiratory flow even at peak ventilation (Figure 3).

## 4 DISCUSSION

By combining electromyography of the diaphragm and extra‐diaphragmatic respiratory muscles, pressure manometry, and CPET parameters, our study provides an integrated physiological assessment of respiratory muscle recruitment during exercise hyperpnea in patients with UDD. This approach allowed us to link altered timing and amplitude of muscle activation with corresponding changes in pressure generation and ventilatory responses, offering a comprehensive picture of compensatory strategies in UDD. The novel finding of our study is that UDD patients adopt distinct compensatory strategies, characterized by earlier recruitment of extra‐diaphragmatic inspiratory muscles and greater expiratory muscle activation, which together support ventilation despite impaired diaphragm function. Importantly, this altered timing of muscle activation was directly linked to pressure data: UDD patients exhibited a greater rise in gastric pressure (Pga) during expiration and relied on earlier activation of the scalene and sternocleidomastoid muscles (with amplitudes comparable to controls) during inspiration to achieve ventilation. In contrast, healthy controls relied more on diaphragm and parasternal intercostal activity at peak. These findings highlight how both the timing and pattern of respiratory muscle recruitment contribute to maintaining high ventilation in UDD.

Previous research has demonstrated that the amplitude of extra‐diaphragmatic muscle activity can increase in response to heightened ventilatory demands, increased respiratory system load, or when breathing occurs at high operational lung volumes, such as near total lung capacity (Campbell, 1955; Raper et al., 1966; Washino et al., 2017). Extra‐diaphragmatic muscles, such as the scalene and sternocleidomastoid, have a greater capacity for rapid contractions compared to the diaphragm and are less affected by the loss of mechanical efficiency at high lung volumes. Regarding the latter, achieving higher lung volumes at end‐inspiration (EILV) is a hallmark of exercise hyperpnea, even in healthy subjects who do not develop dynamic hyperinflation (i.e., do not increase end‐expiratory lung volume; EELV) during exercise. The diaphragm experiences a decrease in mechanical efficiency when operating at high lung volumes, primarily due to the shortening of diaphragmatic muscle fibers, moving them away from their optimal length‐tension relationship (De Troyer et al., 2009; De Troyer & Boriek, 2011; De Troyer & Wilson, 2009). These changes have a comparatively smaller impact on the mechanical efficiency of extra‐diaphragmatic inspiratory muscles.

Peak inspiratory flow, a surrogate of contraction velocity, and tidal volume relative to inspiratory capacity were similar between UDD patients and controls. Because residual volume was not available in controls, absolute EELV and EILV could not be determined, limiting assessment of their influence on muscle activity. Instead, dynamic IC changes were reported, which reflect operational lung volumes if total lung capacity remains constant during exercise (O'Donnell et al., 2001). Under this assumption, absolute changes in diaphragm and extra‐diaphragmatic muscle length were likely comparable between groups.

Peak ventilation was ~10% lower in UDD patients which seems meaningful even though this difference did not reach statistical significance. After adjusting Borg scores for peak ventilation, UDD patients showed a higher probability of reporting abnormally high dyspnea, accompanied by a distinct muscle activation pattern. They exhibited greater expiratory muscle activity during expiration, reflected by a larger Pga rise, which likely assisted the diaphragm in the subsequent inspiration. In contrast, scalene and sternocleidomastoid EMG activity was similar in amplitude to controls but occurred earlier. These findings align with animal models of diaphragm paralysis, indicating that while expiratory muscles partly compensated for diaphragm dysfunction, early recruitment of extra‐diaphragmatic inspiratory muscles remained critical for achieving ventilation. Together, increased expiratory and extra‐diaphragmatic inspiratory muscle activity allowed UDD patients to sustain relatively high ventilation (up to 70% MVV), underscoring their compensatory role in maintaining ventilation despite diaphragm weakness.

Therefore, we observed that while increased expiratory muscle activity may partially compensate for a dysfunctional diaphragm, the role of extra‐diaphragmatic inspiratory muscles remains crucial during periods of increased ventilatory demand. Earlier activation of the scalene and sternocleidomastoid muscles may be an adaptation to diaphragm dysfunction. A similar pattern occurs during inspiratory muscle training (IMT) (Rodrigues et al., 2020), which strongly recruits extra‐diaphragmatic muscles beyond levels seen in exercise hyperpnea (Rodrigues et al., 2020). This may explain the benefits of IMT in patients with persistent diaphragm dysfunction, by improving efficiency and fatigue tolerance of these muscles.

IMT is used to improve respiratory muscle function in various clinical populations (Ammous et al., 2023; Azambuja et al., 2020; Caicedo‐Trujillo et al., 2023; Katsura et al., 2015). In the context of UDD, our findings suggest that IMT may be particularly relevant as a means of strengthening extra‐diaphragmatic inspiratory muscles, which play a compensatory role when diaphragm function is impaired. Indeed, some studies show that clinical benefits of IMT in UDD occur without measurable improvements in diaphragm contractility (Rodrigues et al., 2020; Schaeffer et al., 2023), pointing instead to enhanced recruitment and conditioning of extra‐diaphragmatic muscles. This aligns with EMG data indicating that IMT preferentially activates these muscles (Rodrigues et al., 2020). Thus, IMT may represent a practical strategy to improve compensatory muscle function in UDD, although future work should clarify optimal training protocols and assess diaphragm versus extra‐diaphragmatic adaptations.

### 4.1 Methodological considerations

Our study incorporates simultaneous measurements of muscle electrical activity, respiratory pressures and ventilatory variables, providing valuable insights into the relationship between these variables. Our sample size is relatively small, likely limiting the statistical power for some variables. Likewise, the fact that we found significant differences in a relatively small sample is an indication that these differences are real. However, the impact of unilateral diaphragm dysfunction is poorly understood due to the small number of studies performed with this population, which highlights the uniqueness of our data set. Nevertheless, it provides an opportunity to understand the impact of “pure” diaphragmatic dysfunction on extra‐diaphragmatic respiratory muscles since our population had no other lung or cardiovascular dysfunction/diseases that could affect breathing mechanics, or respiratory muscle activity.

We defined EMG onset as a 5% increase above baseline activity. While breaths with larger peak EMG could appear later by this method compared with visual detection, our algorithm has been validated and shows strong agreement with manual analysis by two independent investigators. Importantly, the algorithm avoids the subjectivity inherent in manual methods, where assessors may differ in judging when EMG activity begins (Rodrigues et al., 2021), and applies standardized criteria across all breaths and muscles, thereby minimizing variability (Rodrigues et al., 2021).

Surface EMG can be influenced by cross‐talk (e.g., parasternal intercostals and pectorals) and movement‐related activity. To limit these effects, we applied standard filters and quantified EMG RMS only during inspiration (Hermens et al., 2000; Jonkman et al., 2024; Rodrigues et al., 2021; Spasojevic et al., 2021), ensuring that signals reflected inspiratory effort rather than movement artifacts. While surface EMG may not isolate a single muscle, it reliably represents inspiratory muscle activation. Importantly, surface EMG of extra‐diaphragmatic respiratory muscles has been widely used in research and linked to clinical outcomes across patient groups (Basoudan et al., 2020; Grasshoff et al., 2021; Jonkman et al., 2024; Koopman et al., 2023; Louvaris et al., 2020; Luiso et al., 2020; Rodrigues et al., 2020, 2024; Schmidt et al., 2013).

EELV and EILV influence contractile effort, diaphragm EMG activity, and recruitment of extra‐diaphragmatic muscles (Niro et al., 2021). Because complete lung function testing was not performed in controls, residual volume was unavailable, limiting absolute quantification of EELV and EILV. In UDD patients, diaphragm weakness raises concern that IC may not represent a true inspiration to total lung capacity. To verify this, we compared transdiaphragmatic pressure during IC maneuvers at rest, iso‐ventilation, and peak. The absence of significant differences supports that UDD patients achieved total lung capacity during serial IC measurements.

Certain procedures, such as phrenic nerve conduction testing, the BDI questionnaire, and supine spirometry, were not performed in the matched control group. However, inspiratory and expiratory muscle strength and exercise capacity measured during CPET were within normal ranges, supporting that the control group provided an appropriate basis for comparison.

In conclusion, patients with unilateral diaphragm dysfunction rely on a different breathing strategy than healthy individuals during high ventilatory demand, compensating for reduced diaphragm function by recruiting expiratory and extra‐diaphragmatic inspiratory muscles earlier and more intensely. These adaptations highlight the potential of targeting extra‐diaphragmatic respiratory muscles in therapies aimed at improving breathing in diaphragm dysfunction.

## AUTHOR CONTRIBTIONS

AR, ZL, and DL were involved in conception of the work, data collection, data analysis, drafting of the manuscript, and critically revised the manuscript for important intellectual content. SD, MCP, and TG were involved in data collection, drafting of the manuscript, and/or critically revised the manuscript for important intellectual content. RG was involved in conception of the work, drafting of the manuscript, and critically revised it for important intellectual content. All authors contributed to drafting of the manuscript and critically revised it for important intellectual content.

## FUNDING INFORMATION

This research was supported by the Research Foundation Flanders (FWO project grant G053721N). AR was supported by a Canadian Institutes of Health Research (CIHR) Fellowship (#187900).

## CONFLICT OF INTEREST STATEMENT

No conflicts of interest, financial or otherwise, are declared by the author(s).

## ETHICS STATEMENT

This study was approved by the Ethics Committee Research of University Hospitals Leuven (S60754). All participants provided written informed consent prior to enrolment in accordance with the Declaration of Helsinki.

## Data Availability

The data that support the findings of this study are available from the corresponding author upon reasonable request.
