# Correction to Pattern of extra‐diaphragmatic respiratory muscle activity during exercise in patients with unilateral diaphragm dysfunction

**DOI:** 10.14814/phy2.70679

**Published:** 2025-11-25

Rodrigues, A., Louvaris, Z., Dacha, S., Pereira, M. C., Gojevic, T., Schaeffer, M. R., Janssens, L., Janssens, W., Reid, W. D., Gayan‐Ramirez, G., Testelmans, D., Brochard, L., Gosselink, R., & Langer, D. (2025). Pattern of extra‐diaphragmatic respiratory muscle activity during exercise in patients with unilateral diaphragm dysfunction. *Physiological Reports*, 13, e70635. https://doi.org/10.14814/phy2.70635

The affiliation for the author “Laurent Brochard” is incorrect. It should instead be as follows:

1. Keenan Centre for Biomedical Research, Li Ka Shing Knowledge Institute, Unity Health Toronto, Toronto, Ontario, Canada

2. Interdepartmental Division of Critical Care Medicine, University of Toronto, Toronto, Ontario, Canada

We apologize for this error.

